# Validation of a UV Spectrometric Method for the Assay of Tolfenamic Acid in Organic Solvents

**DOI:** 10.1155/2015/216249

**Published:** 2015-12-10

**Authors:** Sofia Ahmed, Nafeesa Mustaan, Muhammad Ali Sheraz, Syeda Ayesha Ahmed un Nabi, Iqbal Ahmad

**Affiliations:** Baqai Institute of Pharmaceutical Sciences, Baqai Medical University, 51 Deh Tor, Toll Plaza, Super Highway, Gadap Road, Karachi 74600, Pakistan

## Abstract

The present study has been carried out to validate a UV spectrometric method for the assay of tolfenamic acid (TA) in organic solvents. TA is insoluble in water; therefore, a total of thirteen commonly used organic solvents have been selected in which the drug is soluble. Fresh stock solutions of TA in each solvent in a concentration of 1 × 10^−4 ^M (2.62 mg%) were prepared for the assay. The method has been validated according to the guideline of International Conference on Harmonization and parameters like linearity, range, accuracy, precision, sensitivity, and robustness have been studied. Although the method was found to be efficient for the determination of TA in all solvents on the basis of statistical data 1-octanol, followed by ethanol and methanol, was found to be comparatively better than the other studied solvents. No change in the stock solution stability of TA has been observed in each solvent for 24 hours stored either at room (25 ± 1°C) or at refrigerated temperature (2–8°C). A shift in the absorption maxima has been observed for TA in various solvents indicating drug-solvent interactions. The studied method is simple, rapid, economical, accurate, and precise for the assay of TA in different organic solvents.

## 1. Introduction

Tolfenamic acid (TA) is a nonsteroidal anti-inflammatory drug (NSAID) that belongs to the family of fenamates and is used in both humans and animals for the management of pain and inflammation [1]. Recently, it has gained tremendous popularity due to its anticancer activity against a variety of cancers [2–7]. TA has also shown potential for use in slowing down the progression of Alzheimer's disease [8–11]. A number of workers have employed various techniques to determine TA in different samples, for example, solutions, milk, serum, blood, and urine [12–22], or to characterize its physicochemical properties [23–28], but still the literature lacks information regarding its analysis in different solvents using a validated method.

TA occurs as a white or slightly yellow crystalline powder and is practically insoluble in water [29, 30]. The official pharmacopoeial assay method for TA involves direct titration against sodium hydroxide solution [29]. Previously, validated methods for the quantitative analysis of TA, both as a pure compound and in tablet dosage form, have been reported using FTIR and UV spectrometry [31]. Both methods showed good accuracy and precision for the assay of TA with the UV method showing comparatively better results. Both techniques were found to be statistically comparable with the official titrimetric method [31].

The present study has been designed to validate the UV spectrometric assay procedure for the analysis of pure TA in different organic solvents according to the guidelines of International Conference on Harmonization (ICH) [32]. TA is a water insoluble drug and such study would provide useful data which would help in its determination with high accuracy and precision in various pharmaceutical systems incorporating organic solvents.

## 2. Materials and Methods

### 2.1. Materials

Tolfenamic acid was purchased from Sigma-Aldrich Company Ltd., Dorset, UK. All the solvents used in this study were of analytical grade having the highest degree of purity. The details of the solvents used in this study are reported in Table 1.

### 2.2. Thin Layer Chromatography (TLC)

TLC was performed to check the purity of TA used in this study according to the method reported in British Pharmacopoeia [29]. The substance (25 mg) was dissolved in a mixture of methanol and methylene chloride (1 : 3, v/v) and diluted to 10 mL with the same mixture and 10 *μ*L of the solution was applied to 250 *μ*m silica gel GF_254_ plates. It was developed with the mobile phase up to 2/3 distance of the plate. The plate was dried and viewed under 254 nm UV lamp (Uvitec, Cambridge, UK).

### 2.3. Ultraviolet Spectrometry

All absorbance measurements and spectral determinations were carried out on a Shimadzu UV-visible spectrophotometer (model UV-1601) using quartz cell of 10 mm path length. The cells were employed always in the same orientation using appropriate control solutions in the reference beam. The baseline correction was made by the built-in baseline memory at the initializing period while auto-zero adjustment was made by one-touch operation. The wavelength scale was also calibrated automatically by the instrument. The instrument was calibrated for the absorbance scale according to the method described in British Pharmacopoeia [29], by using 0.057–0.063 g/lit of potassium dichromate in 0.005 M sulfuric acid.

The absorbencies of the corresponding series of solutions in each solvent were measured against a reference of the same solvent in the region of 250–400 nm. Quartz cells were closed with a cap to prevent evaporation of the organic solvent during absorbance measurements.

### 2.4. Preparation of Stock and Test Solutions for Validation Studies

The stock solutions of TA for validation studies were prepared in a concentration of 1.0 × 10^−4 ^M (2.62 mg%) in the individual solvent (Table 1). The stock solutions were thoroughly stirred each time by the aid of a magnetic stirrer for 30 min. During stirring the solutions were kept in a tightly closed container to avoid evaporation of the organic solvent. The test solutions in each solvent were prepared from the stock by making appropriate dilutions in the concentration range of 1.0–8.0 × 10^−5 ^M. The stock solutions and the respective dilutions were found to be completely transparent in appearance. Each time fresh solutions were prepared. The solutions were protected from light and the absorbance was then measured immediately. All experiments were performed in triplicate.

### 2.5. Validation of the Analytical Method

The UV method for the assay of TA was validated according to the guidelines of ICH [32]. Different parameters of validation for TA were studied which are described as follows.

#### 2.5.1. Linearity and Range

The linearity of the method was determined by preparing calibration curves of absorbance versus the concentration of TA of the test solutions in the concentration range of 1.0–8.0 × 10^−5 ^M for each solvent. The linearity was statistically determined by regression analysis of five concentrations used in triplicate. The linearity range was selected on the basis of absorbance values in the region of around 0.2–0.8. This range of absorbance is known to provide values with the highest precision [34]. The molar absorptivity and A (1%, 1 cm) values were also determined from the calibration curve.

#### 2.5.2. Accuracy

The accuracy of the proposed method was determined by adding known concentrations of the drug in the solutions followed by their analysis by the UV spectrometric method. Three different concentrations in triplicate from the studied range were selected and analyzed for the recovery.

#### 2.5.3. Precision

The precision of the developed method was calculated by performing nine determinations at three concentrations covering the specified range. The precision was determined by calculating relative standard deviation (%RSD) of the mean recoveries.

#### 2.5.4. Limit of Detection (LOD) and Limit of Quantitation (LOQ)

LOD and LOQ of the developed method were calculated from the standard deviation of the *y*-intercept and slope of the calibration curve using the following formulae:

(1) LOD=3.3×σS,LOQ=10×σS,

where *σ* is the standard deviation of the intercept and *S* is the slope of the calibration curve.

#### 2.5.5. Robustness

The robustness of the method was determined by studying small changes in the assay wavelength (±2 nm). This parameter was studied thrice in the similar range used for the determination of TA (i.e., 1.0–8.0 × 10^−5 ^M). The accuracy and precision of the method were determined.

#### 2.5.6. Solution Stability

The stability of stock solutions of TA was studied at room (25 ± 1°C) and refrigerated temperature (2–8°C). The stock solutions of TA were prepared in pure solvents at a concentration of 1 × 10^−4 ^M (2.62 mg%). The samples were stored in tightly sealed glass containers protected from light. A 5 mL aliquot of the sample was taken each time and the absorbencies were measured at 0-, 1-, 2-, 3-, and 24-hour time interval.

## 3. Results and Discussion

### 3.1. Confirmation of Purity of Tolfenamic Acid

In order to study spectrometric characteristics of a compound, it is necessary to confirm the purity of the material to avoid any effect on the position and intensity of the absorption maxima as well as on the validation of the assay method. In the case of TA a thin layer chromatography (TLC) examination was conducted to detect any spots other than that of TA on TLC plates. The TLC test for TA has been carried out according to the method described in British Pharmacopoeia [29]. TA appeared as a single spot confirming the purity of the material.

### 3.2. Nature of Solvents and Spectral Characteristics of Tolfenamic Acid

The use of solvents in UV-visible spectrometric measurements depends on the nature of the compound to be characterized or analyzed. The solvent must be transparent in the region in which the compound exhibits absorption spectrum. The compound should have enough solubility to obtain a reasonably clear absorption spectrum. It is also important to consider any possible interaction of the solvent with the absorbing molecule to impart a shift in the absorption maxima. It has been reported that polar solvents such as water, alcohols, esters, and ketones (containing lone pair of electrons) tend to obscure vibrational spectra. The nonpolar solvents such as cyclohexane, chloroform, and benzene give spectra somewhat similar to that of a gas (better band resolution) [35]. The maximum absorption wavelength of the absorption band depends on the degree of solute-solvent interaction and the nature of solvent [36–39]. The solvent dependent spectral shifts arise from either nonspecific (dielectric enrichment) or specific (e.g., hydrogen bonding) solute-solvent interactions. Considering the interactions between the solute and solvent molecule and the intensity of these interactions, a change in the absorption spectrum of the molecule (e.g., *λ*_max_ and *ϵ*_max_) can be expected. Such a change has been described as solvatochromism [40]. The organic solvents have a different polar character as indicated by the dielectric constant of the medium. It has been observed that an electronic transition of a compound may lead to a modification of the charge distribution by the solvent used. This would result in some change in the position and intensity of the absorption maxima depending on the nature of the solvent. The extent of solute-solvent interaction would give an indication of the type of electronic transition undergone by the molecule [41].

The lower wavelength limit of common solvents in the UV and visible spectra strongly depends on the purity of the solvent (Table 1). For example, ethanol and the hydrocarbon solvents are frequently contaminated with benzene which absorbs below 280 nm [35]. Therefore, the highest/spectroscopic grade solvents should always be used for the measurement of the absorption spectra of organic compounds; otherwise the true spectral characteristics of a compound may not be obtained due to the presence of interfering impurities.

**Figure 1:**
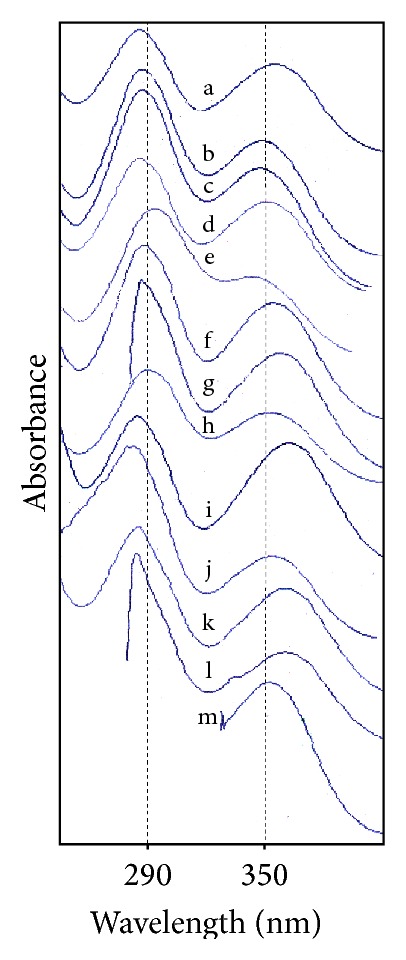
Variations in the absorption maxima of TA in organic solvents. (a) Acetonitrile, (b) methanol, (c) ethanol, (d) 1-propanol, (e) 1-butanol, (f) 1-hexanol, (g) benzyl alcohol, (h) 1-octanol, (i) dichloromethane, (j) ethyl acetate, (k) chloroform, (l) toluene, and (m) acetone.

The spectral characteristics of TA including the value of absorption maxima, respective molar absorptivities (*ϵ*), and specific absorbance [*A* (1%, 1 cm)] in various organic solvents are reported in Table 2. A consideration of the values of absorption maxima of TA in various organic solvents shows that their *λ*_1max_ range from 286 to 294 nm and *λ*_2max_ from 332 to 354 nm (Figure 1, Table 2) with regression values (*R* ^2^) of 0.99905–0.99988 showing very small scatter of the points around the calibration curves (Table 3).

Similarly, a variation in the values of *ϵ*_max_ in these solvents is also observed (Table 2). This is probably due to the degree of interaction between the solute and the solvent to cause a shift in the absorption maxima with accompanying change in the intensity of absorption as indicated by the values of *ϵ*_max_. The high values of *ϵ*_max_ indicate *π*-*π* ^*∗*^ electronic transition in the molecule. The values of *ϵ*_*λ*_1__ range from 7930 to 10960 M^−1 ^cm^−1^ and those of *ϵ*_*λ*_2__ from 5310 to 8967 M^−1 ^cm^−1^.

### 3.3. Validation of the Assay Method

The UV spectrometric assay of TA in various solvents has been validated according to the guidelines of ICH [32], including the following parameters.

#### 3.3.1. Linearity

**Figure 2:**
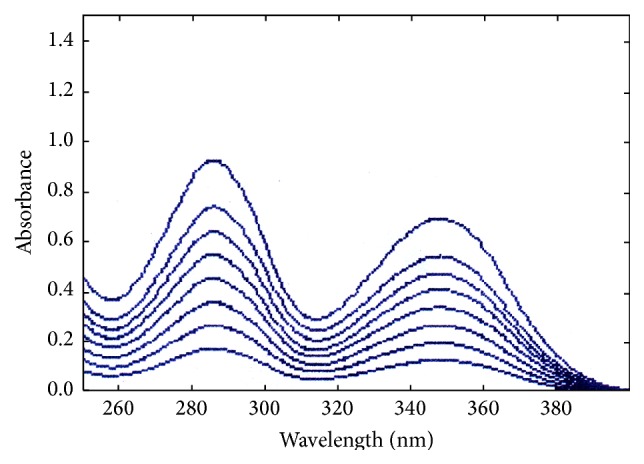
Overlay spectra of TA in acetonitrile.

Linearity determines the ability of the method to obtain the results that are directly proportional to the concentration of the analyte within a given range by plotting a calibration curve. TA is 2-[(3-chloro-2-methylphenyl)amino]benzoic acid and gives two peaks in the region of 280–360 nm (Figure 1). The short wavelength peak in the region below 300 nm is more prominent with a greater intensity than the one present above 300 nm. Therefore, calibration curves of TA in each solvent have been prepared with respect to the short wavelength peak in the majority of solvents (Table 2). On the contrary, in the solvent that showed some interference or has a cutoff point near or above the prominent peak of TA such as acetone (330 nm), benzyl alcohol (282 nm), and toluene (286 nm), it has been assayed and validated with respect to the long wavelength peak (Figure 1). Although the calibration curves in benzyl alcohol and toluene have been prepared with respect to the short wavelength peak due to their interfering cutoff points they have further been validated for TA assay using the long wavelength peak. A linear relationship has been found for TA in each solvent and the statistical data are reported in Table 3. The intercept values are significantly close to zero in each case thus confirming the peak purity of TA. The overlay spectra of TA in acetonitrile are shown in Figure 2.

#### 3.3.2. Range

It is defined as the interval between the upper and lower concentrations of the analyte that have been demonstrated to be determined with acceptable precision, accuracy, and linearity. The absorbance values in the range of 0.2–0.8 are known to offer the highest precision [34]. Therefore, similar pattern has also been followed in this study in determining the range of TA in each solvent. The ranges for the assay of TA in each solvent are reported in Table 3 which corresponds well to the points in calibration curves.

#### 3.3.3. Accuracy

The accuracy of an analytical method is defined as the degree to which the determined value of an analyte in a sample corresponds to the true value. The results for the percent recovery of TA in different organic solvents are reported in Table 3. Although the results show good accuracy for TA in each solvent comparatively the mean recovery in 1-octanol followed by ethanol and methanol is better than that of the others due to minimum standard deviations. The standard deviations are small in all cases indicating that the method can be used with high accuracy for the determination of TA in the studied organic solvents.

#### 3.3.4. Precision

Precision of an analytical method is the closeness of agreement between a series of measurements obtained from multiple samples of the studied drug under prescribed conditions. The results for the precision of the method for the assay of TA in various solvents are reported in Table 3. These indicate that the %RSD in the majority of cases is less than 2% and is minimum in the case of 1-octanol with nearly the same values in ethanol and methanol. Thus the studied method is highly reliable for the assay of TA in different solvents.

#### 3.3.5. LOD

It is the lowest concentration of an analyte in a sample that can be detected but not necessarily quantified. It is considered as limit test that indicates that the analyte is above or below a certain value which is usually expressed as percentage of the analyte in the sample. The LOD of TA in each solvent is reported in Table 3. The minimum detection limit of 1.97 × 10^−6 ^M (0.05 mg%) has been found in 1-octanol while the highest of 6.47 × 10^−6 ^M (0.17 mg%) has been found in 1-butanol. This indicates that the UV spectrometric technique is highly sensitive for the detection of TA in various organic solvents.

#### 3.3.6. LOQ

The LOQ determines the lowest concentration of an analyte in a sample that can be quantified with acceptable precision and accuracy under the documented operational conditions of the drug being assayed. The minimum quantification limit of 5.98 × 10^−6 ^M (0.16 mg%) has been found in 1-octanol while the highest of 1.96 × 10^−5 ^M (0.51 mg%) has been found in 1-butanol. The values of LOQ of TA in each solvent are reported in Table 3. All solvents have been found to correspond well with the quantification of TA by UV spectrometric technique indicating that the method is accurate and precise for its assay.

#### 3.3.7. Robustness

The robustness of an analytical method is a measure of its capacity to obtain acceptable results when perturbed by small but deliberate variations. It is basically an indicator of method suitability and reliability during normal use. Absorbance of a solution is dependent upon wavelength, solvent, pH, and temperature. Therefore, these parameters should remain constant throughout the course of the analysis; otherwise significant errors may arise in the quantitative analysis of the samples [34]. In the present study, the reliability of the method has been tested by determining the absorption maxima in each solvent and by changing the assay wavelengths at room temperature (25 ± 1°C). The results showed that small changes in the wavelength of absorption maxima do not affect the accuracy and precision of the assay of TA (Table 4). This indicates that the method is robust under the studied conditions in the majority of the solvents. The highest robustness has been found in acetonitrile whereas the lowest has been found in chloroform followed by acetone (Table 4).

#### 3.3.8. Solution Stability

The solution stability is a measure of the extent to which the studied drug is stable in a solvent being used for the assay over a particular period of time under specified conditions. It is an essential requirement that the analyte should not undergo any chemical change and should remain stable in the particular solvent [34].

The study of TA was carried out at room temperature (25 ± 1°C) and refrigerated temperature (2–8°C). The consistency in absorbance indicated the stability of TA solutions. In all solvents no significant change has been observed in the absorbance of TA after 24 hours of storage either at room temperature or in a refrigerator. However, in spite of the stability of TA in the organic solvents for at least 24 hours, fresh solutions were used for the validation study.

## 4. Conclusion

The present study has employed thirteen commonly used solvents for the validation of a UV spectrometric method for the determination of TA. The results indicated that the method is accurate, precise, robust, economical, and rapid for the assay of TA with a stock solution stability of 24 hours in each solvent. TA exhibits two peaks in the UV region of 280–360 nm. The major short wavelength peak is in the region of 285–295 nm that showed good results for the assay of TA. Those solvents that have a cutoff point in this region or interfere with the major peak can also be used for the determination of TA with respect to the minor long wavelength peak in the region of 335–355 nm.

The results of this study highlight the effect of different solvents on the spectral characteristics of organic molecules of pharmaceutical importance. Some shifts in the absorption maxima of TA have been noted probably due to drug-solvent interaction while absorptivity constants of TA in each solvent have also been determined. These shifts can affect the wavelengths used for the assay of a compound and, therefore, it is necessary to use a particular solvent for assay purpose. It is also necessary to confirm the purity of the solvent used for assay and its interference in the spectral region of the compound to be studied. A detailed investigation of the effect of solvent parameters on the spectral characteristics of a compound is required to develop an understanding of the changes observed. Such study would help the pharmaceutical formulators and analysts to determine TA in pharmaceutical systems incorporating organic solvents.

## Figures and Tables

**Table 1:** List of solvents used for the validation of TA assay in the order of decreasing polarity.

S. number	Name	Purity (%)	Supplier	Dielectric constant^a^
(1)	Acetonitrile	99.9%	VWR	36.64
(2)	Methanol	99.8%	Merck	33.00
(3)	Ethanol	99.8%	BDH	25.30
(4)	Acetone	99.0%	Merck	21.01
(5)	1-Propanol	99.5%	Merck	20.80
(6)	1-Butanol	>99.0%	Merck	17.84
(7)	1-Hexanol	98.0%	Scharlau	13.03
(8)	Benzyl alcohol	>99.0%	Scharlau	11.92
(9)	1-Octanol	99.5%	Merck	10.30
(10)	Dichloromethane	99.8%	Lab-Scan	8.93
(11)	Ethyl acetate	99.0%	Merck	6.08
(12)	Chloroform	99.5%	Merck	4.81
(13)	Toluene	99.5%	Tedia	2.38

^a^[33].

**Table 2:** Absorption maxima and molar absorptivities of TA in organic solvents.

Solvent	Absorption maxima (nm)		Molar absorptivity (M^−1^ cm^−1^)		*A* (1%, 1 cm)	
	*λ*_1max_	*λ*_2max_	*ϵλ*_1max_	*ϵλ*_2max_	*λ*_1max_	*λ*_2max_
Acetonitrile	286	343	9083	6960	352	266
Methanol	289	335	10371	6328	431	242
Ethanol	289	338	10100	6030	390	230
Acetone	—	347	—	8781	336	336
1-Propanol	288	343	10849	7512	438	287
1-Butanol	294	332	10029	5310	384	203
1-Hexanol	289	344	7926	5380	300	205
Benzyl alcohol	289	352	9386	7711	359	295
1-Octanol	290	343	9118	5884	348	225
Dichloromethane	287	350	10956	8967	416	343
Ethyl acetate	287	343	8995	6593	353	252
Chloroform	288	351	8929	4620	338	176
Toluene	288	354	8539	7141	326	273

**Table 3:** Analytical parameters for the determination of TA in various organic solvents.

Solvent	Linearity range, conc. M × 10^−4^ (mg%)^a^	Corr. coeff.	Slope	SE^b^ of slope	Intercept	SE^b^ of intercept	SD^c^ of intercept	Recovery range (%)^d^	Accuracy (%)^e^ ± SD^c^	Precision (%RSD)^f^	LOD^g^ M × 10^−6^ (mg%)	LOQ^h^ M × 10^−5^ (mg%)
Acetonitrile	0.2–0.9 (0.52–2.36)	0.99985	9.08 × 10^3^	0.00419	−0.00102	0.00385	0.01089	98.73–101.07	99.90 ± 0.7899	0.7907	3.96 (0.10)	1.20 (0.31)
Methanol	0.2–0.8 (0.52–2.09)	0.99994	1.04 × 10^4^	0.00273	0.03714	0.00277	0.00734	99.24–100.87	99.99 ± 0.6113	0.6113	2.34 (0.06)	7.08 (0.19)
Ethanol	0.2–0.8 (0.52–2.09)	0.99992	9.43 × 10^3^	0.00281	0.00218	0.00286	0.00757	99.06–100.85	99.96 ± 0.5969	0.5971	2.65 (0.07)	8.03 (0.21)
Acetone	0.3–1.0 (0.79–2.62)	0.99956	8.78 × 10^3^	0.00690	−0.06210	0.00764	0.02021	98.15–101.40	100.11 ± 1.2665	1.2651	7.60 (0.20)	2.30 (0.60)
1-Propanol	0.2–0.8 (0.52–2.09)	0.99971	1.08 × 10^4^	0.00613	0.02508	0.00624	0.01651	98.93–101.18	99.92 ± 0.8370	0.8377	5.02 (0.13)	1.52 (0.40)
1-Butanol	0.2–0.8 (0.52–2.09)	0.99953	1.00 × 10^4^	0.00730	0.00595	0.00743	0.01966	95.92–102.39	99.76 ± 2.0754	2.0805	6.47 (0.17)	1.96 (0.51)
1-Hexanol	0.3–1.0 (0.79–2.62)	0.99984	7.93 × 10^3^	0.00378	−0.01175	0.00418	0.01107	98.78–100.99	100.05 ± 0.7348	0.7344	4.61 (0.12)	1.40 (0.37)
Benzyl alcohol	0.2–0.8 (0.52–2.09)	0.99983	9.39 × 10^3^	0.00413	0.02471	0.00421	0.01113	99.15–101.27	99.95 ± 0.7502	0.7506	3.91 (0.10)	1.19 (0.31)
1-Octanol	0.2–0.8 (0.52–2.09)	0.99996	9.12 × 10^3^	0.00203	0.01139	0.00206	0.00546	99.11–100.71	100.01 ± 0.5566	0.5565	1.97 (0.05)	5.98 (0.16)
Dichloromethane	0.2–0.8 (0.52–2.09)	0.99992	1.10 × 10^4^	0.00328	−0.05499	0.00334	0.00884	99.03–101.31	100.06 ± 0.7268	0.7264	2.66 (0.07)	8.07 (0.21)
Ethyl acetate	0.2–0.8 (0.52–2.09)	0.99987	8.99 × 10^3^	0.00338	−0.00287	0.00344	0.00911	98.88–101.69	99.96 ± 0.8841	0.8845	3.34 (0.09)	1.01 (0.26)
Chloroform	0.2–0.8 (0.52–2.09)	0.99976	8.93 × 10^3^	0.00465	0.02979	0.00473	0.01252	98.88–101.30	99.95 ± 0.8556	0.8560	4.63 (0.12)	1.40 (0.37)
Toluene	0.2–0.9 (0.52–2.36)	0.99990	8.54 × 10^3^	0.00312	−0.00004	0.00287	0.00811	98.67–101.32	100.10 ± 0.8768	0.8760	3.13 (0.08)	9.50 (0.25)

^a^ *n* = 3.

^b^SE = standard error.

^c^SD = standard deviation.

^d^Recovery (%) = (amount found/amount added) × 100

(amount found = absorbance − intercept/slope).

^e^Accuracy (%) = mean recovery range.

^f^% of relative standard deviation (%RSD) = (SD/mean) × 100.

^g^Limit of detection (LOD) = 3.3 × (SD of intercept/slope).

^h^Limit of quantification (LOQ) = 10 × (SD of intercept/slope).

**Table 4:** Robustness of the proposed method in different organic solvents.

Solvents	Wavelength (*λ*_max_ ± 2 nm)^a^	Accuracy (%) ± SD	Precision (%RSD)
Acetonitrile	284 288	100.02 ± 0.4554 100.03 ± 0.5216	0.4553 0.5215

Methanol	288 292	99.91 ± 0.7512 99.88 ± 1.0125	0.7519 1.0137

Ethanol	288 292	99.93 ± 0.7414 99.87 ± 1.2148	0.7420 1.2164

Acetone	345 349	100.29 ± 2.8852 100.33 ± 3.3030	2.8769 3.2920

1-Propanol	286 290	99.90 ± 0.9924 99.69 ± 2.4774	0.9934 2.4851

1-Butanol	292 296	99.73 ± 2.1447 99.99 ± 1.4541	2.1506 1.4543

1-Hexanol	287 291	100.06 ± 0.9026 100.05 ± 0.8686	0.9021 0.8681

Benzyl alcohol	287 291 350 354	99.90 ± 1.1057 99.94 ± 0.7845 100.14 ± 1.4184 100.14 ± 1.4571	1.1068 0.7850 1.4165 1.4550

1-Octanol	288 292	99.91 ± 0.7796 99.89 ± 0.9320	0.7803 0.9330

Dichloromethane	285 289	100.16 ± 1.3225 100.18 ± 1.4535	1.3203 1.4508

Ethyl acetate	285 289	99.82 ± 1.8881 99.84 ± 1.7719	1.8914 1.7748

Chloroform	286 290	99.66 ± 3.1248 99.67 ± 3.1182	3.1353 3.1285

Toluene	286 290 352 356	99.81 ± 1.3132 100.10 ± 0.8523 100.04 ± 0.5126 100.04 ± 0.5611	1.3157 0.8514 0.5124 0.5608

^a^ *λ*_max_ for each solvent are reported in Table 2.
